# Clinical utility of the BIWACO score for patients with atrial fibrillation after percutaneous coronary intervention

**DOI:** 10.1007/s00380-022-02128-6

**Published:** 2022-07-23

**Authors:** Teruki Takeda, Tomohiro Dohke, Yoshiki Ueno, Toshiki Mastui, Masanori Fujii, Tomoyuki Takayama, Kenichi Dochi, Akashi Miyamoto, Hiroshi Mabuchi, Atsuyuki Wada

**Affiliations:** 1 Division of Cardiology, Koto Memorial Hospital, Higashiomi-shi, Japan; 2 Division of Cardiology, Kohka Public Hospital, Koka-shi, Japan; 3 Division of Cardiology, Nagahama Red Cross Hospital, Nagahama-shi, Japan; 4 Division of Cardiology, Shiga Hospital JCHO, Otsu-shi, Japan; 5 Department of Cardiology, Omi Medical Center, 1660 Yabase, Kusatsu-shi, Shiga 525-8585 Japan

**Keywords:** Anticoagulants, Atrial fibrillation, Percutaneous coronary intervention, Risk score

## Abstract

No predictive clinical risk scores for net adverse clinical events (NACE) have been developed for patients with atrial fibrillation (AF) after percutaneous coronary intervention (PCI). We evaluated NACE to develop clinically applicable risk-stratification scores in the Bleeding and thrombotic risk evaluation In patients With Atrial fibrillation under COronary intervention **(**BIWACO) study, a multicenter survey which has enrolled a total of 7837 patients. We also investigated the current status and time trends for the use of antithrombotic drugs. A total of 188 AF patients who had received PCI were examined. At discharge, 65% of patients were prescribed a triple therapy (TT), 6% were prescribed a dual therapy, the remaining 29% of patients received dual-antiplatelet therapy. After 4 years, the fraction of patients continuing TT decreased by 15%, whereas oral anticoagulant alone was only 2% of patients. NACE developed in 20% of patients, resulting in death in 5% of the patients, and the remaining 13% experienced bleeding events. We developed risk scores for NACE comprising the five strongest predictive items, which we designated BIWACO scores. The area under the curve was 0.774 for NACE. Our study explored the differences in treatment practices and guideline recommendations for antithrombotic therapy. We concluded that our BIWACO score is useful for predicting clinical outcomes in AF-patients after PCI.

## Introduction

Atrial fibrillation (AF) is a major cause of stroke, heart failure (HF), sudden death, and cardiovascular morbidity, and requires oral anticoagulant (OAC) therapy to mitigate thromboembolic events [1]. Coronary artery disease is present in 20–30% of patients with AF, and approximately 5–10% of patients requiring percutaneous coronary intervention (PCI) present with AF, including chronic OAC [2–4]. Since dual-antiplatelet therapy (DAPT) is recommended for all stent-implanted patients, those with AF undergoing PCI are at a substantially higher risk of serious bleeding from the use of multiple antithrombotic drugs. Thus, it is very important to select the types of antithrombotic agents to use, after carefully considering the balance between safety and efficacy. A network meta-analysis of randomized controlled trials investigated the safety and efficacy of different antithrombotic regimens post–PCI for these high-risk patients with AF. The study concluded that an antithrombotic regimen of a direct antagonist oral anticoagulant (DOAC) plus P2Y12 inhibitor results in less bleeding compared with a regime of vitamin K oral antagonist (VKA) plus DAPT [5]. Therefore, current guidelines recommend a short period of triple therapy (TT) (i.e., an OAC plus aspirin and a P2Y12 inhibitor) followed by dual therapy (DT) comprising an OAC and one antiplatelet agent [6, 7]. The general consensus is to continue oral anticoagulation and to modify antiplatelet drug intensity and duration as necessary. However, TT or DT are likely to be continued for longer periods in daily clinical practice reflecting physicians’ concerns about thrombotic safety. Thus, there seems to be a disconnect between current antithrombotic regimens in practice and the recommendations in the guidelines. Little is known about trends in the actual use of the individualized antithrombotic therapies in patients with AF undergoing PCI [8]. Several risk scores for thrombosis and bleeding have been proposed previously for patients with either PCI or AF [9–12], but no predictive clinical risk factor scores have been established for patients with AF and concomitant PCI. Considering that 2nd generation drug-eluting stents (DES) cause a lower incidence of stent thrombosis compared to previous stents [13], we evaluated the outcomes of antithrombotic therapy in terms of the rates of net adverse clinical events (NACE) in the Bleeding and thrombotic risk evaluation In patients With Atrial fibrillation under COronary intervention (BIWACO) study cohorts. We secondary aimed to develop simple, clinically applicable risk-stratification scores. We also investigated the current status and trends over time for the use of DAPT and OAC. These aims clarify how long antithrombotic regimens are applied to AF patients after PCI in daily practice in Japanese AF patients with stent placement.

## Methods

### Study design

The BIWACO study was a prospective, multicenter, observational registry endeavor designed to provide up to 48 months of clinical follow-up to facilitate the evaluation of the different characteristics and outcomes of antiplatelet and anticoagulant therapy after PCI in patients with AF. The study was performed from January 2014 to December 2017 in the Shiga prefecture, which has the largest ancient lake Biwako in Japan, during which time consecutive patients with PCI therapy for any coronary artery disease indication was enrolled from five institutions. AF in patients undergoing PCI was defined as either a history of AF or AF occurring during the hospital stay. The BIWACO study was registered with the University Hospital Medical Information Network-Clinical Trials Registry (UMIN-CTR, no. UMIN 00,002,694, R000030644). Inclusion criterion for the registry were patients (≥ 20 years of age) with AF (paroxysmal, persistent, or permanent AF) and any coronary artery disease after 2nd generation coronary stent implantation. AF was documented on 12-lead electrocardiogram or Holter monitoring. Patients were included who had been treated with an oral anticoagulant (warfarin or a DOAC; dabigatran, rivaroxaban, apixaban, and edoxaban) and antiplatelet agents (aspirin, clopidogrel and prasugrel). In patients receiving warfarin, international normalized ratio (INR) was ≥ 1.6 at the time of study entry according to the Japanese guidelines. Patients were excluded for the following reasons: cardiogenic shock or hypotension, defined as systolic blood pressure < 90 mmHg; vulnerable ischemic stroke and any active bleeding, current HF hospitalization, a history of stent thrombosis, coexisting active tumor, poorly controlled hypertension, severe infections, severe liver injury, and pregnancy. Patients who are scheduled to undergo surgical procedures or plain balloon angioplasty were also excluded. The participating institutions comprised five cardiovascular centers (Omi Medical Center, Koto Memorial Hospital, Kohka Public Hospital, Nagahama Red Cross Hospital and Shiga Hospital JCHO). This study was carried out in accordance with the Declaration of Helsinki and the Ethics Committee of Omi Medical Center approved the protocol (No. 2020-0004). Patients provide written informed consent before being enrolled. The interventional strategy and stent selection were left to the discretion of the operator in all procedures.

### Status of the use of antithrombotic drugs

Anticoagulant and antiplatelet status was assessed after PCI and followed until the end of the study. We defined OAC as warfarin, dabigatran, rivaroxaban, apixaban, and edoxaban. Dose selection of each DOAC was evaluated based on the manufacturer labeling recommendations in Japan. Time in the therapeutic range was not incorporated into this analysis, because we checked the international normalized ratio (INR) only at enrollment and could not evaluate its lability. Antiplatelet drugs were aspirin, clopidogrel, and prasugrel. The DAPT duration was left to the discretion of the operator and the family doctor. During the follow-up period, we noted any changes in the use of antithrombotic drugs.

### Endpoints

The primary endpoints for this study were to investigate composite outcomes of NACE defined as the composite of all-cause death, thrombotic, and bleeding events within 4 years of the PCI procedure. An important secondary endpoint was to develop clinically applicable risk-stratification scores. Cardiac death included sudden death, progressive HF, and fatal myocardial infarction. HF was diagnosed if the patient had a history of hospitalization for HF or if the left ventricular ejection fraction was < 40%. Chronic kidney disease (CKD) was diagnosed if there was persistent proteinuria or if the estimated glomerular filtration rate (eGFR) was < 60 mL/min/1.73 m^2^. Bleeding was defined as the occurrence of Bleeding Academic Research Consortium type 2, 3, or 5 and complications were defined as the requirement for blood transfusion or prolonged hospitalization owing to subcutaneous hematoma, gastrointestinal bleeding, or intracranial bleeding [14]. Thrombotic events were defined as the sudden onset of a focal neurologic deficit in a location consistent with the territory of a major cerebral artery and an acute vascular occlusion of a coronary and peripheral arteries confirmed by angiography. In this study, we also evaluated the current status and trends over time for the use of anticoagulant and antiplatelet therapy in patients with AF after PCI.

### Measurements

Clinical baseline characteristics included age, sex, height, body weight, blood pressure, past medical history, smoking status, use of concomitant drugs, and the information concerning PCI lesion, procedure and coronary stents. Red and white blood cell counts, hemoglobin, hematocrit, platelet count, aspartate transaminase, alanine aminotransferase, γ-glutamyl transpeptidase, creatine kinase, blood urea nitrogen, creatinine, blood glucose, hemoglobin A1_C_, and brain natriuretic peptide were measured locally at baseline.

### Statistical analysis

Continuous variables are reported as mean ± standard deviation. We compared baseline clinical characteristics by the presence or absence of NACE using Student t tests and chi-square tests for continuous and categorical variables, respectively. We constructed a multivariable Cox proportional hazards regression for time to first occurrence of NACE over 4 years of follow-up as the outcome. We cautiously chose candidate variables while referring to previous studies [9–11] and clinical experience to avoid overfitting in a multivariable analysis. The observed event rate was calculated as a Kaplan–Meier estimate of time to first event and differences were assessed with the log-rank test. Candidate variables for the model were chosen from the list of variables remaining significant at a threshold *p* value of < 0.05. To create the simple BIWACO score, we retained 5 predictors from the full model with metrics of discrimination (Harrel’s C statistic). Point values were assigned to each predictor equally. Discrimination was by calculating the area under the curve (AUC) and was expressed as the C statistic. We employed a nonparametric approach to the comparison of the areas under ROC curves between two studies. A *p* value of < 0.05 was considered statistically significant in all analyses.

## Results

### Study population

A total of 7837 consecutive patients who received PCI with DES implantation were enrolled in the BIWACO study. The first registration began April 1, 2014 (the registration number 2014-001). Of these, only 219 (2.8%) had a medical history of AF at the time of hospitalization. We excluded 31 patients because they were treated by balloon angioplasty alone, leaving 188 patients in this analysis. The median follow-up period was 794 days (interquartile range, 504–1362 days). Baseline characteristics are listed in Table 1. Mean age was 74.2 years, and139 (73.9%) study participants were males. Second-generation DES were used in all patients, of whom 28.2% underwent PCI for acute coronary syndrome (ACS), and 14.9% had HF with reduced ejection fraction. A history of ischemic stroke or bleeding was present in 17.6% and 8.6% of patients, respectively, as shown in Table 1.

**Table 1:** Overall Characteristics of patients with or without NACE

Characteristics	All, *n* = 188	NACE (–), *n* = 151	NACE ( +), *n* = 37	*P* value
Age, y	74.2 ± 9.7	73.3 ± 9.8	77.9 ± 8.3	0.010
Male, *n* (%)	139 (73.9)	115 (76.2)	24 (64.9)	0.16
BMI, kg/m^2^	23.1 ± 4.0	23.5 ± 4.0	21.4 ± 3.4	0.004
Permanent AF, *n* (%)	86 (46.0)	68 (45.3)	18 (48.7)	0.72
Hypertension, *n* (%)	160 (85.1)	126 (83.4)	34 (91.9)	0.20
Diabetes mellitus, *n* (%)	67 (35.8)	51 (34.0)	16 (43.2)	0.29
ACS, *n* (%)	53 (28.2)	42 (27.8)	11 (29.7)	0.82
HFrEF (LVEF < 40), *n* (%)	28 (14.9)	18 (11.9)	10 (27.0)	0.02
CKD (eGFR < 60), *n* (%)	112 (59.6)	83 (55.0)	29 (78.4)	0.009
Anemia, *n* (%)	85 (45.2)	62 (41.1)	23 (62.2)	0.021
Prior PCI, *n* (%)	70 (37.2)	53 (35.1)	17 (46.0)	0.22
Prior CABG, *n* (%)	12 (6.4)	9(6.0)	3 (8.1)	0.63
Prior ischemic stroke, *n* (%)	33 (17.6)	20 (13.3)	13 (35.1)	0.002
Prior major bleeding, *n* (%)	16 (8.6)	11 (7.3)	5 (13.9)	0.20
Prior hemorrhagic stroke, *n* (%)	3 (1.6)	2 (1.3)	1 (2.7)	0.55
Platelet count (× 10^4^/μL)	19.3 ± 5.7	19.3 ± 5.4	19.3 ± 6.7	0.97
HbA1c (%)	6.4 ± 1.3	6.3 ± 1.4	6.4 ± 1.0	0.74
LDL-C (mg/dL)	98.7 ± 28.1	99.4 ± 27.8	96.1 ± 29.7	0.53
Stent diameter, mm	3.04 ± 0.44	3.05 ± 0.46	3.00 ± 0.36	0.63
ACEIs/ ARBs, *n* (%)	115 (61.2)	88 (58.3)	27 (73.0)	0.10
Diuretics, *n* (%)	75 (40.0)	54 (35.8)	21 (56.8)	0.02
Statins, *n* (%)	100 (53.2)	83 (55.0)	17 (46.0)	0.32
PPIs, *n* (%)	122 (64.9)	98 (64.9)	24 (64.9)	0.99
VKA, *n* (%)	57 (30.3)	38 (25.2)	19 (51.4)	0.002
DOACs, *n* (%)	77 (41.0)	66 (43.7)	11 (29.7)	0.12
Triple therapy, *n* (%)	122 (64.9)	95 (62.9)	27 (73.0)	0.25
Dual therapy, *n* (%)	12 (6.4)	9 (6.0)	3 (8.1)	0.16
DAPT without OAC, *n* (%)	54 (28.7)	47 (31.1)	7 (18.9)	0.25

Data given as *n*, mean ± SD, median (IQR) or *n* (%). *NACE* net adverse clinical events; *BMI* body mass index; *ACS* acute coronary syndrome; *HFrEF* heart failure with reduced ejection fraction; *CKD* chronic kidney disease; Anemia, defined as hemoglobin level < 13 g/dl in men and < 12 g/dl in women; *PCI* percutaneous coronary intervention; *CABG* coronary artery bypass grafting; *LDL-C* low density lipoprotein cholesterol; *PPIs* proton pump inhibitors, *VKA* vitamin K antagonist; *DOACs* direct oral anticoagulants; *DAPT* dual antiplatelet therapy

### Clinical outcomes by NACE

**Fig. 1:**
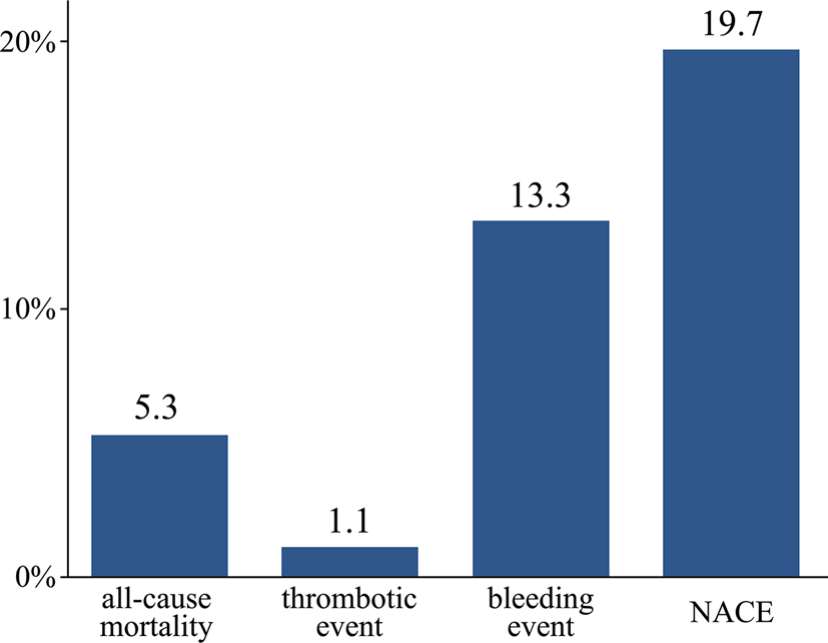
Percentage of Incidence. *NACE* net adverse clinical events

Overall characteristics of patients with NACE (*n* = 37, 19.7%) or without NACE (*n* = 151, 80.3%) at baseline are listed in Table 1. Over 4 years, 37 patients developed NACE, including 10 (5.3% of all patients) who died and 2 with thrombotic events. The remaining 25 (13.3%) experienced bleeding events, as shown in Fig. 1. Causes of death of the 10 deceased patients were cardiac (*n* = 3), stroke (*n* = 4), systemic thrombosis (*n* = 1), and non-cardiovascular (*n* = 2). Major bleeding was observed in 25 patients including one intracranial hemorrhage and 13 patients with severe gastrointestinal bleeding. There were differences in the incidence of major bleeding between patients on TT (*n* = 15), DT (*n* = 5), or DAPT (*n* = 5), but these differences were not statistically significant. Patients who experienced NACE were older, had lower body mass index (BMI), and had a higher prevalence of HF, previous ischemic stroke, renal dysfunction, and anemia relative to those without NACE.

### Predictors of integer risk scores (the BIWACO risk score)

**Fig. 2:**
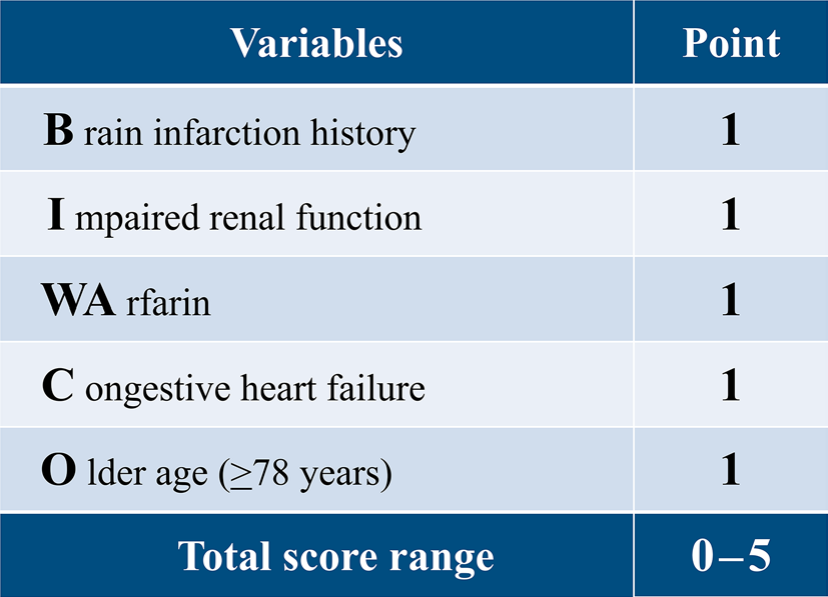
The predictive risk scores (BIWACO scores) for NACE. *NACE* net adverse clinical events

**Fig. 3:**
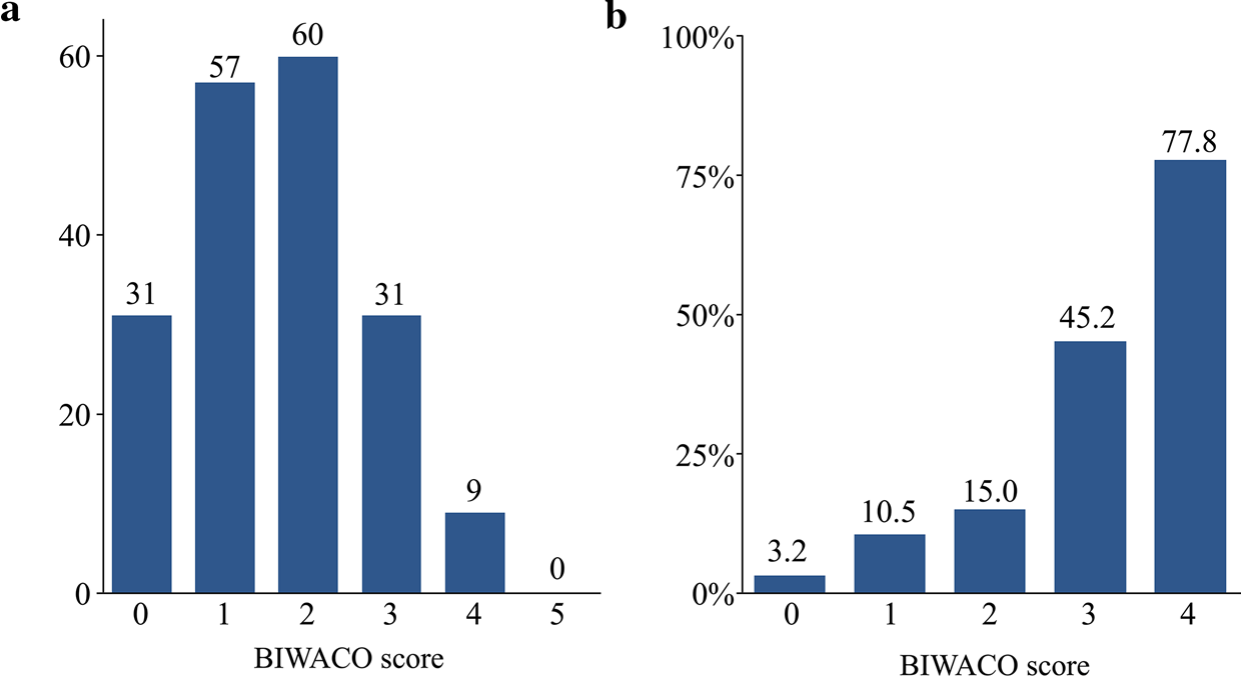
Distribution of the BIWACO score. **a** Patient number according to the each BIWACO score (point 0–5). **b** Percentage of event rates according to the each BIWACO score

**Fig. 4:**
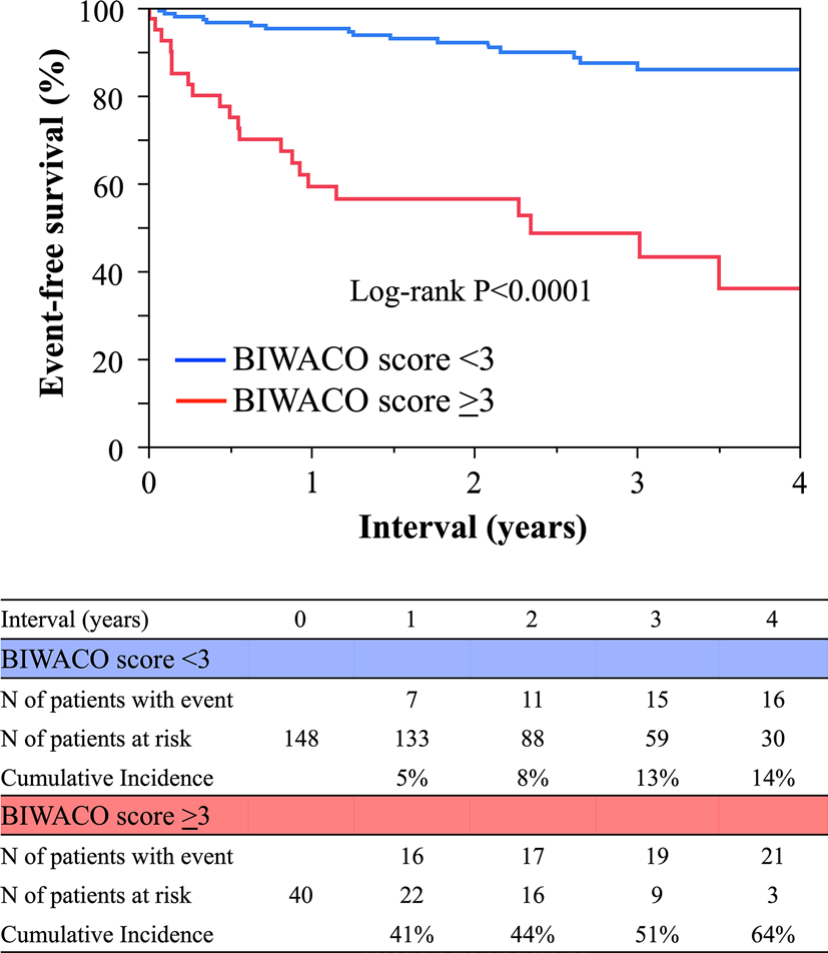
Kaplan–Meier estimates of patients free of NACE among patients stratified by high (red line)-vs-low (blue line) BIWACO risk scores. *NACE* net adverse clinical events

**Fig. 5:**
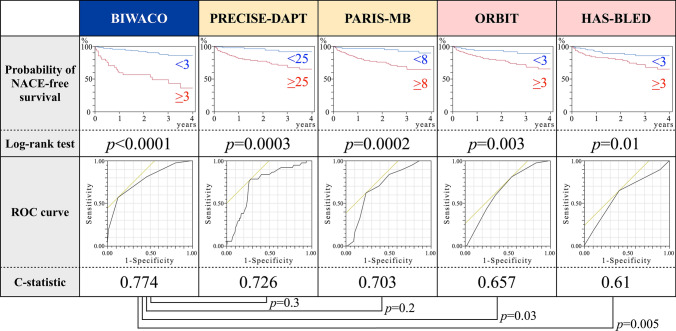
Comparison of Kaplan–Meier curves and AUC for predicting NACE among patients stratified according to BIWACO, PRECISE-DAPT, PARIS-MB, ORBIT, or HAS-BLED scores. *AUC* area under the curve; *NACE* net adverse clinical events

As shown in Table 2, we listed the several statistically significant variables while referring to the previous predictive studies [9, 11]. The BIWACO study included the following independent predictors of NACE: age, BMI, HF with reduced ejection fraction, previous ischemic stroke, chronic kidney disease (CKD), anemia, and prescription of VKA. From the multivariable model, we developed a simple, numerical risk score for NACE from the five strongest predictive items as follows: brain infarction history; Impaired renal function [estimated glomerular filtration rate (eGFR) < 60 mg/dL/1.73 m^2^]; WArfarin use; Congestive HF (EF < 40%); and Older (age ≥ 78 years), yielding the score acronym “BIWACO” from the BIWACO study (Fig. 2). We assigned point values equally based on the log scale because of the magnitude of the modelling coefficient representing each variable’s association with NACE. The distribution of the BIWACO score ranged from 0 to 5, in which the peak AUC was at 3 points (AUC 0.77) as shown in the Fig. 3. After stratifying patients according to low (BIWACO score, 0–2, *n* = 148) or high (BIWACO score, 3 or greater, *n* = 40) categories, we constructed NACE-free curves of events stratified by the best cut-off value for the BIWACO score within each estimated risk group, as shown in Fig. 4. Figure 5 presents a comparison of C-statistics (95% confidence intervals) for score discrimination of four different risk scoring systems, namely, the PARIS-MB, PRECISE-DAPT, ORBIT, and HAS-BLED scores. Using this analytical approach, the AUC value of the BIWACO study was 0.774, indicating that our model showed the best discrimination, followed by the PRECISE-DAPT, the PARIS-MB score, the ORBIT score, and, finally, the HAS-BLED score.

**Table 2:** Univariate and multivariate analysis for NACE

Variables	Univariate			Multivariate		
	HR	95% CI	*P* value	HR	95% CI	*P* value
Age	1.06	1.02–1.10	0.005	collinearity with Age ≥ 78		
Age ≥ 78	2.69	1.40–5.35	0.003	2.16	1.01–4.79	0.048
Male	0.60	0.31–1.27	0.15	0.93	0.45–1.99	0.84
BMI	0.86	0.78–0.95	0.002	collinearity with BMI < 22		
BMI < 22	2.39	1.25–4.70	0.009	1.39	0.65–2.99	0.40
Current smoker	0.80	0.35–1.63	0.55			
Diabetes Mellitus	1.29	0.66–2.46	0.45			
Hypertension	2.00	0.72–8.31	0.21			
HFrEF	2.47	1.14–4.95	0.024	2.64	1.18–5.49	0.020
Previous ischemic stroke	3.08	1.52–5.96	0.002	2.91	1.39–5.85	0.006
CKD	2.89	1.38–6.78	0.004	2.38	1.11–5.69	0.025
Anemia	2.29	1.19–4.57	0.013	1.10	0.51–2.42	0.82
Triple therapy	1.59	0.79–3.44	0.20			
VKA	2.53	1.32–4.86	0.005	2.31	1.16–4.65	0.018
DOACs	0.63	0.30–1.23	0.18			
Previous major bleeding events	1.72	0.59–4.05	0.29			

*HR* hazard ratio; *CI* confidence interval; *BMI* body mass index; *HFrEF* heart failure with reduced ejection fraction; *CKD* chronic kidney disease; Anemia, defined as hemoglobin level < 13 g/dl in men and < 12 g/dl in women; *VKA* vitamin K antagonist; *DOACs* direct oral anticoagulants

### Changes in antithrombotic treatment regimen

**Fig. 6:**
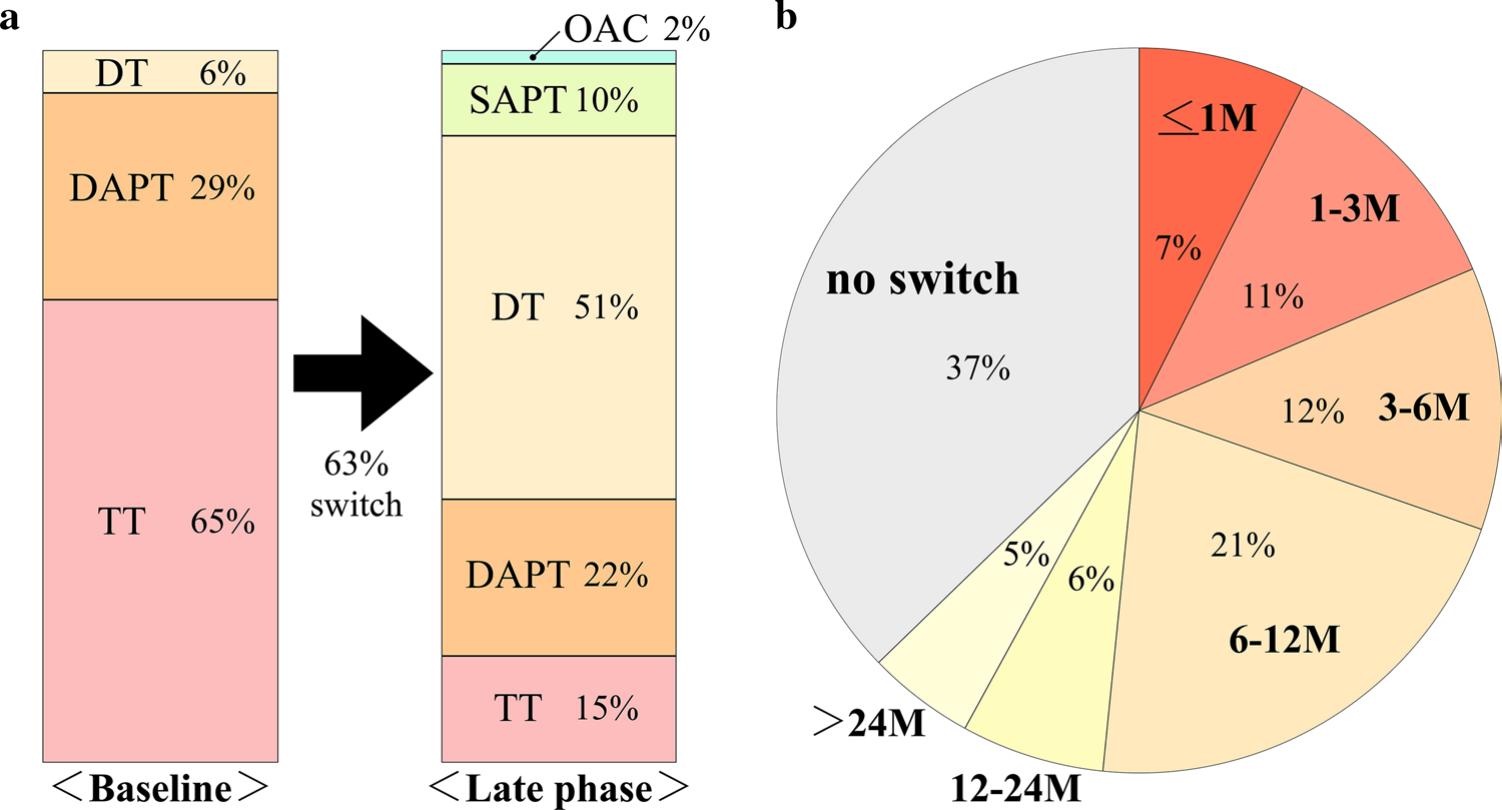
Antithrombotic drug prescriptions and switching regimens. **a** Trends in prescriptions of antithrombotic regimens from baseline to the late phase. The figure shows the proportion of patients receiving single antiplatelet therapy (SPAT), oral anticoagulant alone (OAC), dual-therapy (OAC plus antiplatelet therapy) (DT), dual antiplatelet therapy (DAPT), or triple therapy (TT). **b** Timing of anti-thrombotic regimen switching

Considering the proportion of patients at discharge, 65% were prescribed TT (*n* = 122), while 6% were prescribed DT (*n* = 12; comprising one OAC in combination with aspirin or a P2Y12 receptor inhibitor) and the remaining patients were on DAPT (*n* = 55, 29%), as shown in Fig. 6a. Warfarin was used as the OAC in 30.3% patients, whereas 40.4% were treated with DOAC and 29.3% were not prescribed any OAC. The antiplatelet drugs used were aspirin (95.2%), clopidogrel (87.2%), and prasugrel (11.2%). During the follow-up period, 63% patients were switched to another drug within 48 months after PCI (Fig. 6b). The number of patients continuing TT decreased by 15%, DT increased by 51%, and DAPT decreased by 22%. Single antiplatelet therapy (SAPT) was used by 10% of patients and OAC alone only by 2% of patients. Of the patients on TT at discharge, 66% were switched to DT, 6% to DAPT, and 5% to SAPT, with 23% of patients continued TT therapy.

## Discussion

In the present study, we surveyed the current clinical landscape concerning the administration of anticoagulant and antiplatelet therapy in Japanese patients with AF undergoing coronary stenting. First, this study explored the treatment practices and NACE outcomes achieved using several different antithrombotic regimens. Second, we developed simple scores to predict risks for NACE. The resulting BIWACO score is a novel risk prediction model for AF patients undergoing PCI that enabled a moderately improved discrimination of events concordant with the previously reported DAPT or AF risk scales. We propose the use of BIWACO scores to identify the most at-risk AF-patients on multiple antithrombotic agents after PCI. Finally, the proportion of patients on TT was higher and the use of aspirin was more common and for a longer duration, with 15% of patients still on TT at the end of the follow-up period.

### Predictive scores for NACE in patients with AF undergoing PCI

For a comprehensive judgment on DAPT duration, the PARIS score or the CREDO-Kyoto risk scores are useful predictors of clinical outcome in daily practice after PCI [9, 15]. However, for AF, additional antiplatelet therapy and PCI themselves are strong characteristic risk factors, thus, we selected patients with AF undergoing PCI and modeled predictive scores for NACE. CKD, HF, prescription of VKA, prior stroke experience, and higher age were found to be independent predictors of thrombosis and bleeding in our study. Among our clinical endpoints, bleeding occurred in 13.3% of patients as the most frequent clinical event. In the current study, 60% of patients who experienced bleeding events were receiving TT. In the WOEST trial, serious bleeding rates were similar to those observed in this study, and were also significantly lower in the DT group than in the TT group (6.5 vs 12.7%: HR, 0.49) [16]. A 2020 network meta-analysis of data from more than 11,000 patients concluded that, compared with TT (as the reference), the odds ratios for thrombosis in myocardial infarction (TIMI) major bleeding were 0.52 for DOAC plus P2Y12 inhibitor [17]. Indeed, among the BIWACO scores, the use of warfarin was an independent predictor of NACE. In the KICS study of 2,481patients undergoing PCI, the presence of prior stroke was reported to increase the recurrence, complications, and mortality rate, which implies that stroke is an independent factor in determining the prognosis [18]. History of prior stroke was common in PCI patients, and the BIWACO score showed that it was associated independently with NACE.

We consider that increasing age has an independent effect on the NACE after stenting in patients with AF [19]. However, previous studies employed an arbitrary cutoff, e.g., > 75 years [20]. A definitive age threshold value of 78 years-old would be preferable for our clinicians. Since maximal AUC was 78 years-old (AUC = 0.64) from our cohort using a calculated ROC curve, we employed 78 years-old as the age threshold in order to define age-related NACE risk and help improve NACE risk stratification.

### Discrimination of the BIWACO risk scores concordant with the previous DAPT or AF risk scales

The discriminatory power (AUC value) of the BIWACO risk scores for NACE was 0.774. Similar AUCs were reported in the PARIS-MB, PRECISE-DAPT, ORBIT, and HAS-BLED studies. Although no single score currently has a particularly accurate ability to predict NACE in patients with AF undergoing coronary stenting, our simple BIWACO scores have high C statistics and simplified identifying patients who may be harmed by continuing their antithrombotic regimens.

### Switching the antithrombotic prescriptions in patients with AF after PCI therapy

Compared with oral anticoagulation therapy alone, the addition of DAPT to OAC therapy results in at least a 2-threefold increase in bleeding complications [7]. The best combinations and numbers of agents, as well as their duration of use for an optimal treatment strategy for patients with ACS and elective PCI and with coexisting AF are still under intensive investigation [21, 22]. Therefore, we secondarily assessed how the antithrombotic treatments changed after PCI therapy. BIWACO registry patients seemed to belong to high risk groups, probably because our registry had many elderly patients and those with low body weights. In a Danish nation-wide registry, within 1 year, bleeding events were recorded in 6.3% of patients. Bleeding rates were 22.6, 20.3, and 14.3 events per 100 person-years for TT, DT, and DAPT, respectively, within 30 days [23]. It has been suggested that a DT regimen (oral anticoagulant plus single antiplatelet therapy with a P2Y12 inhibitor) instigated at hospital discharge should be considered for most patients, whereas extending the use of aspirin beyond hospital discharge should be considered only for selected patients at high ischemic/thrombotic risk, but low bleeding risk and for a limited period of time [21]. After the release of DOAC, four randomized trials and meta-analyses using DOAC consistently showed a 20–40% reduction in bleeding events in the DOAC DT groups as compared with the TT groups, without increasing ischemic events [16, 24–26]. The findings of those RCTs engendered a paradigm shift from triple to dual therapy. TT should be given only for the periprocedural period within 2 weeks, followed by DT with a DOAC and clopidogrel, according to the Japanese guidelines [27]. In the current study, we evaluated the switching of drug prescriptions after discharge. At first, 65% of the patients were prescribed TT and 51% of patients were switched to DT in the follow-up period. Of the patients on the TT regimen, 66% were switched to DT, however, 23% of patients continued TT. These results suggest that there is discordance between the guidelines and current clinical practice. Even though in the era of 2^nd^ generation stents, clinical trials have already shown that low-risk patients who receive contemporary stent technologies do not need prolonged DAPT to avoid stent thrombosis [28], but some clinicians still seem to fear the occurrence of ischemic events after stent placement.

### Trends of timing the switch of anti-thrombotic therapy

Next, we evaluated trends of timing the switch to a different anti-thrombotic therapy after discharge. We found that only 7% of patients had their regimens changed within one month of stent placement and 37% of patients were not changed at all. The SHINANO registry reported a similar trend as our study in that OACs were administered to only 60% of the AF patients who underwent coronary stenting, which had not changed at 1 year after PCI, even in the second-generation DES era [29]. After 12 months in AF patients with stable coronary artery disease, the AFIRE trial clearly demonstrated that the use of monotherapy with DOAC was not inferior to DT for the rate of the primary efficacy end point including death from any cause and was superior to major bleeding [30]. However, in our study at 1 year after stenting, OAC alone was only 2% of patients. This could be due to the fact that our cardiologists have not recognized the potential effects of DOAC on the pathobiology of coronary artery disease and embolic events in stable AF patients treated with DES [31].

### Anticoagulants at the time of enrollment

We also examined the percentage of patients taking various different anticoagulants at the time of enrollment in the registry. Whereas 30.3% of patients were warfarin users, a DOAC was used by 40.4%, the majority of whom used Xa inhibitors (29.2%) and 11.2% used thrombin inhibitors. A North American expert consensus recommends that a DOAC rather than a VKA should generally be preferred for most patients in the absence of any contraindications [32]. As mentioned above, serious hemorrhages can occur very early after the beginning of treatment and this kind of risk persists over time.

### Study limitations

It has been reported that approximately 20% of patients with ACS and 5–10% of patients undergoing elective PCI have concomitant AF [2, 3, 30]. However, the number of enrolled patients with AF after PCI in our registry was much smaller than originally designed because of slow enrollment. Therefore, one limitation of the current study is that our registry was not specifically powered to detect the risk scores differentially in the individual components of the bleeding and thrombotic events. Of our 2nd generation DES recipients, only 2.8% had AF, that, the prevalence was too low to evaluate any potential associations between different regimens and cardiovascular death and bleeding. The risk estimates may be unreliable if the multivariable date contain too few outcome events relative to the number of independent variables. We cautiously chose candidate variables while referring to those previous studies and clinical experience to avoid overfitting in a multivariable analysis. A second limitation is there was no independent validation database to confirm our BIWACO score. However, the results of our trial will be useful in guiding the selection of OACs and duration of therapy with DAPT. A third limitation is that data on time spent in the therapeutic range of warfarin were only collected for patients at enrollment, and, therefore, we could not evaluate the appropriateness of warfarin therapy during the study. Fourth, as we included patients from 2014 to 2017, improvements in stent technology and antithrombotic therapy results in a discrepancy between our study’s prescription trend and current guidelines in antithrombotic therapy.

## Conclusions

We should change clinical practice to minimize the duration of TT as much as possible and make patient-by-patient decisions on antithrombotic therapy depending on the balance of the individual bleeding and ischemic risks. Under these circumstances, our 5-element BIWACO risk score can provide a simple tool to predict NACE in patients with AF undergoing PCI.

## Abbreviations

OAC: Oral anticoagulant
DAPT: Dual-antiplatelet therapy
DOAC: Direct antagonist oral anticoagulant
VKA: Vitamin K oral antagonist
TT: Triple therapy
DT: Dual therapy
NACE: Net adverse clinical events
CTE: Coronary thrombotic events
MB: Major bleeding
SAPT: Single antiplatelet therapy
